# Physician preferences for non-metastatic castration-resistant prostate cancer treatment

**DOI:** 10.1186/s12894-020-00631-4

**Published:** 2020-06-22

**Authors:** Sandy Srinivas, Ateesha F. Mohamed, Sreevalsa Appukkuttan, Marc Botteman, Xinyi Ng, Namita Joshi, Erica Horodniceanu, A. Reginald Waldeck, Stacey J Simmons

**Affiliations:** 1 Stanford University Medical Center, Palo Alto, California USA; 2 Bayer U.S. LLC, Whippany, NJ USA; 3 Pharmerit International, Bethesda, MD USA

**Keywords:** Prostatic neoplasms, Castration-resistant, Choice behavior, Risk assessment, Physicians

## Abstract

**Background:**

Recent approvals of second-generation androgen receptor inhibitors (SGARIs) have changed the treatment landscape for non-metastatic castration-resistant prostate cancer (nmCRPC). These SGARIs have similar efficacy but differ in safety profiles. We used a discrete choice experiment to explore how United States physicians make treatment decisions between adverse events (AEs) and survival gains in nmCRPC, a largely asymptomatic disease.

**Methods:**

Treating physicians (*n* = 149) participated in an online survey that included 14 treatment choice questions, each comparing 2 hypothetical treatment profiles, which varied in terms of 5 safety and 2 efficacy attributes. We described safety attributes (fatigue, skin rash, cognitive problems, falls, and fractures) in terms of severity and frequency, and efficacy attributes (overall survival [OS] and time to pain progression) in terms of duration of effect. We used a random parameters logit model to estimate preference weights and importance scores for each attribute. We also estimated the amount of survival gain physicians were willing to trade for a reduction in specific AEs between treatment options.

**Results:**

Physicians placed more importance on survival than on time to pain progression, and viewed a reduction in cognitive problems from severe to none, a reduction in risk of a serious fracture from 8% to none, and a reduction in fatigue from severe to none as the most important safety attributes. Physicians were willing to forego 9.1 and 6.6 months of OS, respectively, to reduce cognitive problems and fatigue from severe to mild-to-moderate. To reduce the risk of a serious fracture from 8 to 5% and 5% to none, physicians were willing to trade 3.9 and 5.3 months of OS, respectively.

**Conclusions:**

Physicians were willing to trade substantial amounts of survival to avoid AEs between hypothetical treatments. These results emphasize the importance of carefully balancing therapies’ benefits and risks to ultimately optimize the overall quality of nmCRPC patients’ survival. Nonetheless, it is noted that the results from the study sample of 149 physicans may not be representative of the viewpoints of all nmCRPC-treating physicians.

## Background

Non-metastatic castration-resistant prostate cancer (nmCRPC) is characterized by biochemical progression (i.e., rising prostate-specific antigen [PSA] after androgen deprivation therapy (ADT) without evidence of detectable disease on conventional imaging [1]. Patients with nmCRPC are in a critical period within the disease’s clinical spectrum, as therapeutic interventions can prevent progression to metastatic disease, in which metastases to the bone, soft tissues, or viscera are associated with higher morbidity and mortality [2, 3]. Patients with nmCRPC are typically asymptomatic [1, 4]. Therefore, another therapeutic goal is to minimize treatment-related adverse events (AEs), which can adversely impact patients’ daily activities or lead to treatment discontinuation. Until recently, the treatment of nmCRPC patients consisted of continued ADT, with or without second-line hormonal treatment or active surveillance using PSA doubling time and imaging [5]. The relative lack of evidence on these strategies’ benefits presents a challenge to clinicians making treatment recommendations [5]. This can be further complicated by patients requesting treatment upon experiencing anxiety due to rising PSA levels [6, 7].

Recent approvals of second-generation androgen receptor inhibitors (SGARIs) have changed the nmCRPC treatment landscape. Large phase 3 randomized clinical trials have demonstrated SGARIs’ benefits, notably their ability to prolong metastasis-free survival (MFS) [8–10]. However, SGARIs may be associated with various AEs and trial evidence suggests that different agents exhibit different safety profiles [8–10]. For example, 3 trials in nmCRPC patients reported rates of fatigue (a common AE among SGARIs) ranging from 12 to 33% [8–10].

In this context, nmCRPC patients, their caregivers, and physicians must balance efficacy with AEs when making treatment decisions. Several studies have elicited prostate cancer patients’ benefit-risk preferences [11–17]. Hauber et al. demonstrated that most patients were willing to trade ≥3 months of survival to avoid bone complications [17]. A more recent study in localized prostate cancer also demonstrated that patients trade between survival and AEs [18]. While the patient perspective is paramount, prostate cancer patients’ treatment decisions are often heavily influenced by physician recommendations [19]. To our knowledge, no study has examined physician preferences for nmCRPC treatment. Therefore, by using a formal discrete choice experiment (DCE) approach following recommended practices [20, 21], we aimed to quantify the benefit-risk preferences that United States (US) practicing physicians associate with nmCRPC and SGARI therapy, and examine the extent to which physicians are willing to trade their nmCRPC patients’ overall survival (OS), to minimize or avoid AEs of interest.

## Methods

### Survey development

The DCE is a common approach used in healthcare research to elicit preferences for treatment characteristics (i.e., attributes) and the willingness to accept trade-offs among these attributes [22]. In a DCE, respondents are presented with a series of recurring questions in which they choose a preferred option from sets of hypothetical medication profiles with systematic variations in the levels of attributes. The attributes included in this DCE (Table 1) were selected based on a literature search and qualitative interviews. The literature search focused on preferences and/or outcomes studies and clinical trials of SGARIs [8–17, 23]. Using the preliminary list of search-identified attributes, individual phone interviews were conducted with 5 nmCRPC-treating physicians, 5 nmCRPC patients, and 5 caregivers of nmCRPC patients to evaluate the relevance from all perspectives. During the interviews, respondents were asked to look at a list of preliminary attributes and levels, rate their importance, and comment on their relevance, meaningfulness and comprehensibility. Verbatim quotes were noted and analyzed qualitatively. Based on the feedback from the interviews and discussion with the clinical experts, a final list of attributes and levels were derived and comprised 2 efficacy-related attributes (OS and time to pain progression [TPP]) and 5 AE-related attributes (frequency or severity of fatigue, skin rash, cognitive problems, risk of serious fall, and risk of serious fracture). This research focused on OS instead of MFS (the primary endpoint in the recent SGARI clinical trials) because the MFS definition varies slightly between trials and is a fairly recent endpoint, whereas OS is assumed to be universally understood by most physicians. The qualitative interviews also verified that physicians, patients and caregivers viewed OS as the most important efficacy attribute.

**Table 1:** Attributes, Attribute Labels and Levels included in the DCE

Attributes	Attribute Labels	Levels
Overall Survival	Prolonging life	• 4 years and an additional 12 months
		• 4 years and an additional 6 months
		• 4 years and an additional 3 months
Time to Pain Progression	Delay in time until pain progresses (develops or worsens)	• 3 years and an additional 12 months
		• 3 years and an additional 6 months
		• 3 years and an additional 3 months
Fatigue	Fatigue (lack of energy)	• None
		• Mild-to-moderate (may affect daily activities)
		• Severe (affects self-care)
Skin Rash	Skin rash	• None
		• Mild-to-moderate (30% of the body or less, may affect daily activities)
		• Severe (more than 30% of the body, requires treatment and affects self-care)
Cognitive Problems	Cognitive problems	• None
		• Mild-to-moderate (may affect daily activities)
		• Severe (affects self-care)
Serious Fall	Risk of a serious fall	• None
		• 5% (5 out of 100 people)
		• 8% (8 out of 100 people)
Serious Fracture	Risk of a serious fracture	• None
		• 5% (5 out of 100 people)
		• 8% (8 out of 100 people)

**Fig. 1:**
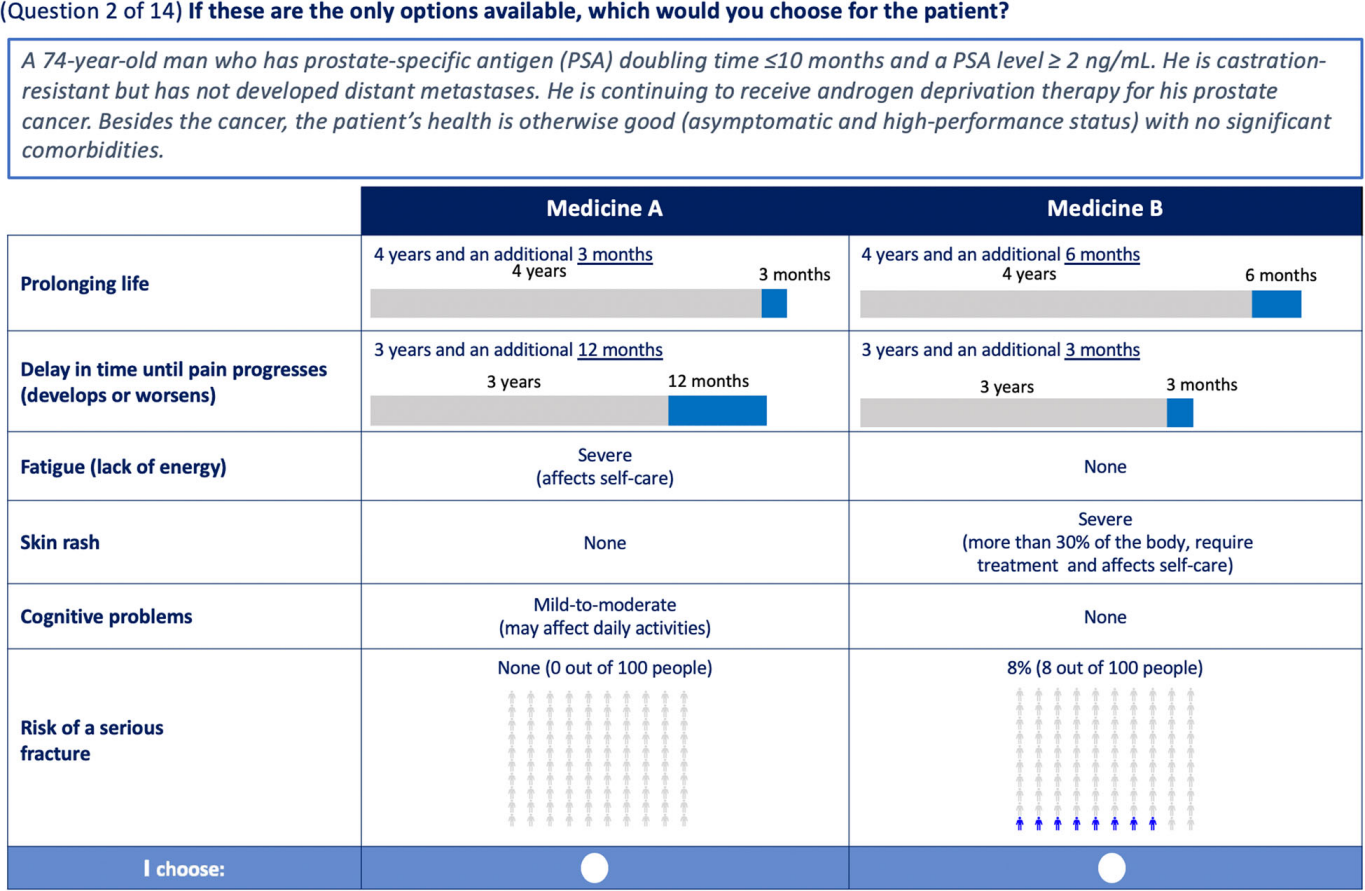
Example of a treatment choice question in the DCE. Note that falls and fractures are correlated and should not be included in the same choice questions. Therefore, risk of a serious fracture was shown in 50% of choice tasks and risk of a serious fall was shown in the other half

To create the alternative choices (i.e., medication profiles), a widely used algorithm (in SAS 9.4) was adapted to create a design that maximized statistical efficiency [24]. The final design was composed of 4 survey versions, each with 14 medication choice questions (i.e., pairs of hypothetical medication profiles; see example in Fig. 1). Each respondent was randomized to 1 of the versions. The survey proceeded as follows: sociodemographic and practice characteristics, treatment attribute descriptions, and 14 medication choice questions. An additional question was added whereby one medication profile was dominant over the other (i.e., higher levels of efficacy and lower levels of AEs) to assess whether respondents were paying attention. Attribute descriptions (appendix Tables 1 and 2, [additional files 1 and 2]) were included before the choice questions to ensure uniformity of interpretation by physicians. Descriptions of AEs and their respective severity levels were adapted from the common terminology criteria for adverse events (CTCAE) [25]. Severe AEs were based on CTCAE grade 3 and above, and mild-to-moderate AEs were based on CTCAE grades 1 and 2. In general, a severe AE affects self-care and a mild-to-moderate AE may affect ability to maintain daily activities.

The initial survey draft was pre-tested via phone interviews with 5 physicians (2 urologists, 3 oncologists) to assess clarity of survey instructions, comprehensibility of attribute descriptions, and whether physicians were willing to make trade-offs amongst attributes. Based on the pre-testing feedback, minor wording changes were made for clarity, and the ranges of levels for 4 attributes (OS, TPP, serious fall, serious fracture) were slightly expanded.

### Study sample

Published preference-related studies in prostate cancer have included sample sizes of 65 to 285 respondents [11, 13–16], and the target sample size of 150 completed responses in this study followed recommendations in DCEs (additional file 3) [26]. The survey was administered online in January 2019 to a convenience sample targeting 75 urologists and 75 oncologists recruited from existing online panels which update their member profiles annually. Eligible respondents were required to have experience treating patients with nmCRPC, be a urologist or oncologist, be ≥18 years of age, and practice within the US. Physicians were excluded if they had participated in an online survey on nmCRPC within the prior 6 weeks, or if they were unwilling to provide consent. All study procedures and materials were reviewed and approved by a centralized US Institutional Review Board.

### Statistical analysis

Descriptive statistics were used to analyze sociodemographic and practice characteristics. Quality checks for DCE responses were evaluated by checking for variability in responses (i.e., respondents who always chose the same medication, such as ‘medication A’, across all 14 choice questions) and assessing the dominance test. Respondents who failed both checks were excluded.

Effects-coded random parameter logit (RPL) models were used to analyze the DCE responses [27]. The RPL model yields a preference weight (i.e., coefficient) for each attribute level, which can be interpreted as the relative preference strength for each attribute level; a greater preference weight indicates a stronger preference [27]. The difference between highest and lowest preference weights (i.e., vertical distance between an attribute’s best and worst levels) is also a measure of the attribute’s overall relative importance over the ranges represented in the DCE [27]. Relative attribute importance scores were also calculated by expressing this vertical distance as a percentage of the total variance observed (i.e., summation of vertical distances across all attributes). The estimated preference weights were used to calculate the rate at which physicians trade between efficacy and treatment risks (i.e., the marginal rate of substitution [MRS]). Specifically, we looked at the amount of OS that physicians would be willing to forego to achieve a given reduction in AE risk or severity. The MRS was calculated as the change in preference weights from a given improvement in AE that could be offset by the change in preference weights from a reduction in OS. In the MRS calculation, we specified OS as a continuous variable, as this was supported by model fit statistics. Separate models were estimated for each physician specialty to examine if there are differences in willingness to trade OS for AE risk/severity reductions. All DCE analyses used STATA/IC 14.2 and R Studio 3.5.0.

## Results

### Sample characteristics

**Fig. 2:**
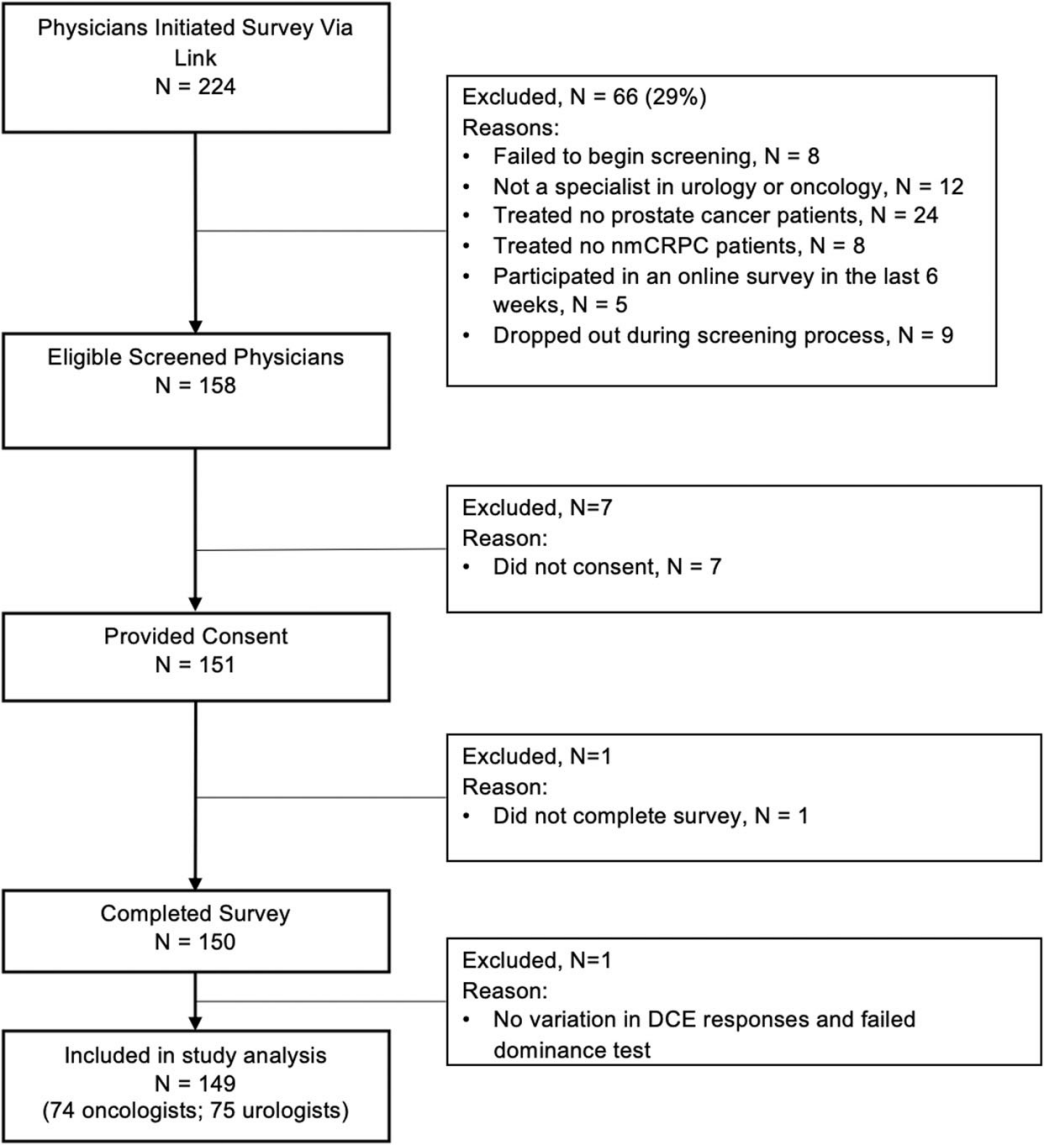
Flowchart of Physician Survey Respondent Selection. Note that the physician who dropped out of the survey dropped out immediately after the first question so no other information was collected. nmCRPC = non-metastatic castration-resistant prostate cancer

Of the 150 physicians who completed the study survey, 1 oncologist was excluded from the analysis due to failing both quality checks (Fig. 2). Respondents’ sociodemographic and practice characteristics are summarized in Table 2. Compared to oncologists, urologists were slightly older and had a higher proportion of males (*P* < 0.05). Among all physicians, the average time in practice was about 17.9 years. The most frequently reported medications prescribed for nmCRPC were enzalutamide (70.5%), leuprolide (67.1%), and bicalutamide (47.0%).

**Table 2:** Characteristics of Physicians and Their Practices by Specialty

Variable	All Physicians (*N* = 149)	Urologists (*N* = 75)	Oncologists (*N* = 74)	*P* value
Age, years				
Mean (SD)	51.27 (10.68)	53.91 (11.27)	48.59 (9.39)	0.004
Gender, n (%)^a^				
Female	15 (10.1)	3 (4.0)	12 (16.2)	0.007
Male	132 (88.6)	72 (96.0)	60 (81.1)	
Race, n (%)^a^				
White	100 (67.1)	54 (72.0)	46 (62.2)	0.474
Black	1 (0.7)	0 (0.0)	1 (1.4)	
Asian	33 (22.1)	13 (17.3)	20 (27.0)	
Middle Eastern or North African	3 (2.0)	2 (2.7)	1 (1.4)	
Native Hawaiian or Other Pacific Islander	1 (0.7)	0 (0.0)	1 (1.4)	
Ethnicity, n (%)^a^				
Hispanic	7 (4.7)	1 (1.3)	6 (8.1)	0.175
Not Hispanic	133 (89.3)	69 (92.0)	64 (86.5)	
Years in practice				
Mean (SD)	17.87 (8.45)	19.52 (8.84)	16.20 (7.75)	0.025
Median (Q1 to Q3)	16.0 (11.0 to 25.0)	19.0 (13.0 to 26.0)	15.0 (10.0 to 21.0)	
Practice type, n (%)				
Office-based private practice	103 (69.1)	55 (73.3)	48 (64.9)	0.291
Hospital-based private practice	18 (12.1)	6 (8.0)	12 (16.2)	
Academic hospital-based	28 (18.8)	14 (18.7)	14 (18.9)	
No. of nmCRPC patients seen/physician/month				
Mean (SD)	26.21 (32.17)	24.08 (29.21)	28.38 (34.98)	0.102
Median (Q1 to Q3)	15.0 (5.0 to 30.0)	12.0 (5.0 to 30.0)	20.0 (6.0 to 35.0)	
Most frequent medications prescribed to treat nmCRPC, n (%)^b^				
Enzalutamide	105 (70.5)	56 (74.7)	49 (66.2)	0.258
Leuprolide	100 (67.1)	61 (81.3)	39 (52.7)	< 0.001
Bicalutamide	70 (47.0)	38 (50.7)	32 (43.2)	0.364
Abiraterone	62 (41.6)	23 (30.7)	39 (52.7)	0.006
Apalutamide	49 (32.9)	28 (37.3)	21 (28.4)	0.245
Goserelin	18 (12.1)	3 (4.0)	15 (20.3)	0.002
Flutamide	15 (10.1)	3 (4.0)	12 (16.2)	0.015
Nilutamide	10 (6.7)	0 (0.0)	10 (13.5)	< 0.001
Triptorelin	6 (4.0)	3 (4.0)	3 (4.1)	> 0.999
Ketoconazole	3 (2.0)	2 (2.7)	1 (1.4)	> 0.999
Histrelin	1 (0.7)	1 (1.3)	0 (0.0)	> 0.999
Others	5 (3.4)	4 (5.3)	1 (1.4)	0.367

^a^ Two (1.3%), 11 (7.4%), and 9 (6.0%) physicians declined to answer questions on gender, race, and ethnicity, respectively

^b^ Physicians were asked to select the 3 most frequent medications that they use to treat nmCRPC patients

### Physician preferences and relative importance of attributes

**Fig. 3:**
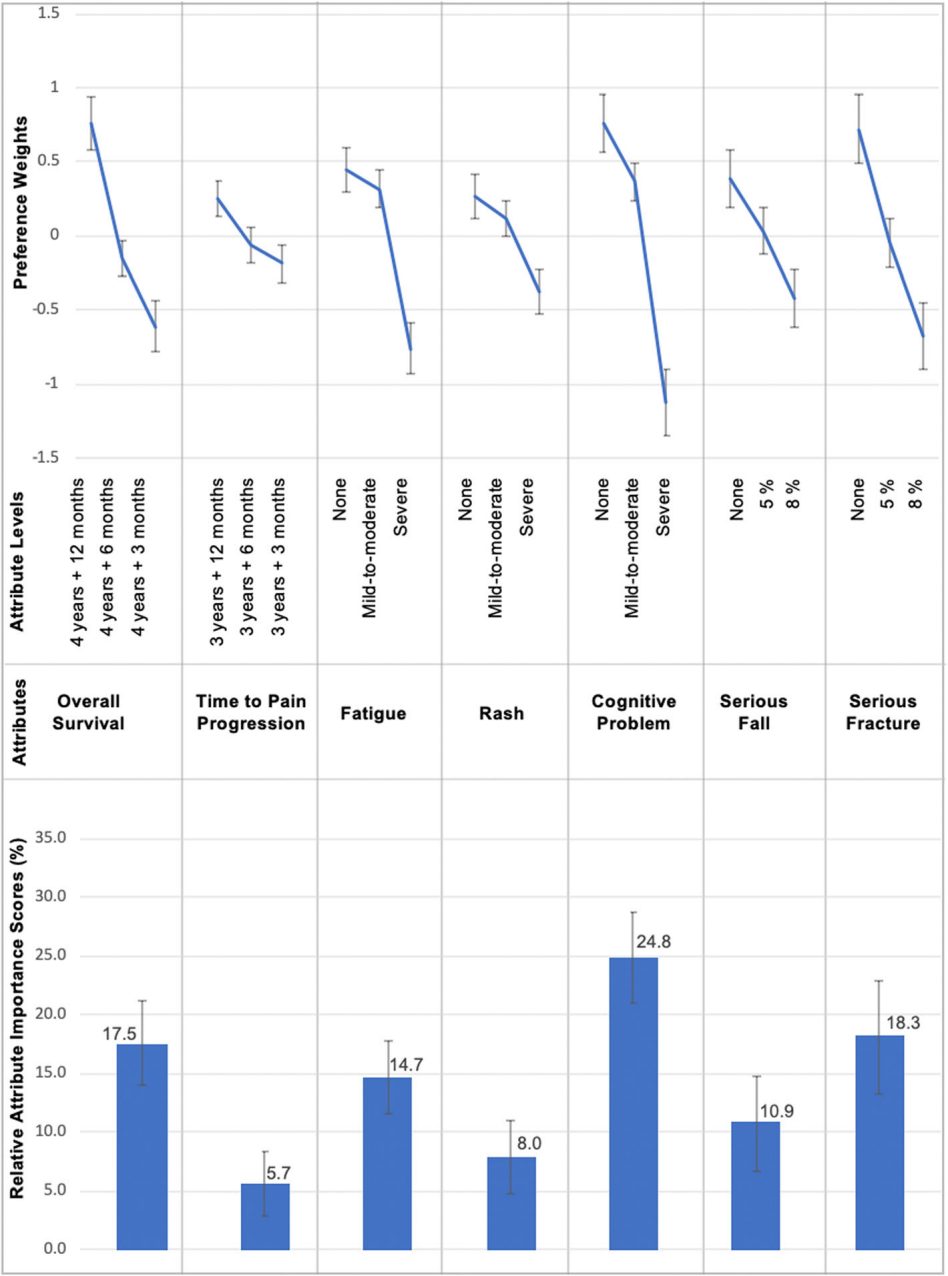
Preference Weights and Relative Importance of Attributes. The line graphs at the top of the figure represent preference weights estimates of each attribute level with 95% confidence intervals (CIs). A more positive preference weight indicates a stronger preference for the level. For example, physicians preferred no fatigue to severe fatigue. The vertical distance between an attribute’s best and worst levels is a measure of the relative importance of the attribute. Relative attribute importance scores (with 95% CI) presented at the bottom of the figure were calculated by expressing this vertical distance as a percentage of the sum of vertical distances across all attributes. The larger the score, the greater the impact that variations in the attribute levels had on treatment choices

The estimated preference weights and relative importance scores are presented in Fig. 3. Physicians’ preferences were naturally and logically ordered (i.e., higher efficacy was preferred over lower efficacy, and lower risks were preferred over higher risks). The difference in preference weights of adjacent levels indicates the relative impact of shifting from one level to the next, where a larger difference signifies a greater impact on treatment choices. The bar graphs in Fig. 3 represent the each attribute’s relative importance to physicians’ treatment choices. Of the efficacy attributes, physicians placed more importance on a 9-month improvement in OS compared to a 9-month improvement in TPP. Physicians viewed the risk attributes in the following order of importance: a reduction in cognitive problems from severe to none; a reduction in risk of a serious fracture from 8% to none; a reduction in fatigue from severe to none; a reduction in risk of a serious fall from 8% to none; and a reduction in rash from severe to none. Of note, physicians valued a reduction in cognitive problems (from severe to none) and serious fractures (from 8% to none) more than a 9-month improvement in OS. For the same amount of risk reduction, physicians placed more importance on avoiding serious fractures compared to serious falls.

### Trade-offs between OS and AEs

Table 3 presents the amount of OS that physicians were willing to forego for various improvements in the other 6 attributes. Specifically, physicians were willing to trade more OS months for AE reductions that they viewed as more important (i.e., cognitive problems, serious fracture, and fatigue). For cognitive problems and fatigue, physicians were on average willing to trade 9.1 and 6.6 months, respectively, to reduce the AEs from severe to mild-to-moderate. To reduce these AEs from mild-to-moderate to none, physicians were willing to trade 2.5 and 0.8 months of OS for cognitive problems and fatigue, respectively, between hypothetical medication profiles. Physicians were willing to trade 3.9 months and 5.3 months of OS, respectively, to reduce the risk of a serious fracture from 8 to 5% and 5% to none. They were also willing to trade 2.9 months of OS to reduce risk of a serious fall from 8 to 5%, and 2.3 months of OS to reduce the risk from 5% to none.

**Table 3:** Number of Months of Overall Survival That Physicians Were Willing to Trade In Return for an Improvement in Other Attributes

	All Physicians		Urologists		Oncologists	
	Reduction, months	95% CI	Reduction, months	95% CI	Reduction, months	95% CI
**Time to pain progression**						
Delay progression from 3 to 12 months	3.0	(1.6, 4.7)	4.1	(1.8, 7.0)	1.5	(−0.4, 3.8)
Delay progression from 6 to 12 months	2.1	(0.8, 3.6)	3.7	(1.6, 6.6)	0.6	(−1.2, 2.6)
Delay progression from 3 to 6 months	0.9	(− 0.5, 2.3)	0.3	(−1.8, 2.5)	0.8	(−1.1, 3.1)
**Fatigue**						
Severe to none	7.5	(5.5, 10.1)	10.6^a^	(7.0, 15.9)	6.2	(3.8, 10.4)
Severe to mild-to-moderate	6.6	(4.9, 9.1)	9.2^a^	(6.3, 13.9)	5.7	(3.3, 9.9)
Mild-to-moderate to none	0.8	(−0.6, 2.3)	1.4	(−1.2, 3.8)	0.5	(− 1.5, 2.4)
**Skin rash**						
Severe to none	4.0	(2.3, 6.0)	7.0	(4.3, 11.0)	1.2	(−1.1, 3.9)
Severe to mild-to-moderate	3.0	(1.7, 4.7)	6.0	(3.5, 9.6)	0.7	(−1.2, 2.8)
Mild-to-moderate to none	0.9	(−0.6, 2.5)	1.0	(−1.3, 3.7)	0.5	(−1.5, 2.7)
**Cognitive problems**						
Severe to none	11.6^a^	(8.9, 15.4)	14.5^a^	(10.2, 21.2)	10.5^a^	(6.7, 17.5)
Severe to mild-to-moderate	9.1^a^	(6.9, 12.1)	12.2^a^	(8.8, 18.0)	7.3	(4.3, 12.5)
Mild-to-moderate to none	2.5	(1.0, 4.2)	2.2	(−0.1, 4.8)	3.2	(0.9, 6.5)
**Serious fall**						
8% to none	5.2	(2.9, 7.9)	4.3	(1.2, 8.2)	6.4	(2.7, 12.1)
8 to 5%	2.9	(1.0, 5.1)	2.4	(−0.5, 5.9)	3.4	(0.7, 7.4)
5% to none	2.3	(0.4, 4.4)	1.8	(−1.0, 5.1)	3.0	(0.2, 6.6)
**Serious fracture**						
8% to none	9.2^a^	(6.4, 12.7)	7.7	(3.7, 12.7)	10.3^a^	(5.9, 17.7)
8 to 5%	3.9	(1.8, 6.3)	3.7	(0.3, 7.4)	4.6	(1.5, 9.0)
5% to none	5.3	(3.1, 7.8)	4.0	(0.7, 7.7)	5.7	(2.7, 10.5)

CI = confidence interval

95% CIs are estimated by simulating 10,000 draws from a multivariate normal distribution defined by the variance-covariance matrix

^a^Estimated months are beyond the range (> 9 months) included in the discrete choice experiment and should be interpreted with caution as it assumes extrapolation beyond the ranges studied

The amount of OS that urologists and oncologists were willing to forego, for their patients, in return for reduction in AEs were similar (95% confidence interval [CI] overlap) except with respect to rash (Table 3). Specifically, urologists placed relatively more importance on avoiding rash compared to oncologists, and were willing to forego about 7.0 months of OS (95% CI: 4.3–11.0) to reduce rash from severe to none, whereas oncologists were not significantly willing to trade OS (CIs cross 0) to reduce rash (Table 3).

## Discussion

This is the first study that systematically evaluates how physicians weigh the benefits versus risks of nmCRPC treatments in the US, focusing on AEs of special interest. New SGARIs, which have demonstrated similar efficacy in terms of delaying metastases, differ in their risk of AEs, which may impact patients’ daily activities and the overall quality of their survival. Our study fills an important gap by considering the types of AEs physicians consider most important, and the extent to which physicians were willing to trade between these AEs and their patients’ OS.

Several key findings emerged from this study. First, we demonstrated that treatment-related AEs greatly influenced physicians’ treatment choices. In fact, physicians appeared to place more importance on avoiding certain AEs (e.g., severe cognitive problems and serious fracture) than improving survival (i.e., 9-month improvement in OS). Although OS is an important treatment goal, physicians were also concerned with minimizing AEs, presumably because nmCRPC patients are largely asymptomatic. Physicians viewed the examined AEs in the following order of importance (most to least): cognitive problems, serious fracture, fatigue, serious fall, and rash. These views were also reflected during the qualitative interviews and pre-tests. Prostate cancer patients are often elderly with multiple comorbidities [28]; as a result, the consequences of increased cognitive problems, fractures, and fatigue can be serious. Understanding how physicians value different treatment attributes (i.e., different AEs and OS) can potentially help them select treatments for a given patient based on the underlying risk for cognitive decline and the treatment’s impact on other important AEs (e.g., fatigue, fractures). These study findings can also potentially inform outcomes for future trials in nmCRPC or decision aids.

Our study also estimated the amount of patient-survival gains that physicians were willing to forego to reduce the risk of treatment-related AEs, particularly those viewed as more problematic. Urologists and oncologists appeared to have different views on the importance of AEs, in particular towards treatment-related rash. Urologists placed more importance on avoiding severe rash and were willing to trade a significant amount OS to avoid rash, as compared to oncologists. However, the study was not designed or powered to detect statistical differences in subgroups, hence these differences should be interpreted cautiously. Other studies have also observed substantial trade-offs between survival and AEs among cancer patients. Bridges et al. found that patients with advanced non-small cell lung cancer require an additional 7.3 months of progression-free survival (PFS) (with mild disease symptoms) to accept an increase in fatigue from none to moderate [29]. Wong et al. found that patients with renal cell carcinoma were willing to trade 4.4 months of PFS to avoid fatigue increasing from mild-to-moderate to severe [30]. Lastly, Uemura et al. observed that patients with castration-resistant prostate cancer prioritized a reduction in fatigue over increasing OS, and de Freitas et al. noted that treatment-related risks were important to patients with biochemical-recurrent prostate cancer [15, 31]. Taken together, these studies further highlight the importance of carefully balancing the goal of extending survival with the introduction of treatment-related AEs.

This study examines treatment preferences in nmCRPC from the physician’s perspective. Understanding physicians’ preferences for treatment choices is important given that treatment decisions in prostate cancer are often heavily influenced by physician recommendations [19]. Undoubtedly, examining benefit-risk preferences from the patient perspective is crucial. A similar DCE has been conducted which showed that nmCRPC patients and caregivers were most concerned with reducing the risks of serious fractures and falls, and the severity of cognitive problems, which is fairly similar with the findings from this physician study [32].

This study is subject to some limitations, one of which is inherent to the nature of DCEs, in that respondents must evaluate hypothetical profiles. However, qualitative work and pre-testing were undertaken to ensure that the treatment profiles were clinically relevant. One other limitation is the inclusion of 5 patients, 5 caregivers and 5 physicians in the qualitative interviews, which may not be representative of viewpoints of all nmCRPC patients and physicians in attribute selection; however, feedback from the interviews were consistent across respondents. The use of online panels for physician recruitment may have also limited the results’ generalizability to the wider nmCRPC-treating physician population, as physicians who participate in online panels may differ from those who do not. In addition, the majority of participating physicians had a private office-based practice and we cannot ascertain if physicians with an academic-based practice would have different preferences. Lastly, the majority of the participating physicians were non-Hispanic white and thus, results might not be generalizable to physicians of other race and ethnicity as benefit-risks preferences can potentially be influenced by cultural background.

## Conclusions

Physicians making treatment decisions for largely asymptomatic patients with nmCRPC were willing to trade substantial amounts of survival to avoid treatment-related AEs of special interest to nmCRPC and SGARI therapy. Physicians were most concerned with minimizing severe cognitive problems, severe fatigue, and serious fractures. However, avoiding lower grade AEs, especially cognitive problems, within nmCRPC is also of clinical relevance. These results underscore the importance of carefully balancing the benefit-risk profiles between newer SGARI therapies when treating this asymptomatic patient population.

## Supplementary information

**Additional file 1: Appendix Table 1**. Labels and Descriptions Used for Efficacy Attributes and Levels

**Additional file 2: Appendix Table 2**. Labels and Descriptions Used for Risk (Adverse Event) Attributes and Levels

**Additional file 3.** Sample Size Considerations in Discrete Choice Experiments

## Data Availability

The datasets during and/or analyzed during the current study available from the corresponding author on reasonable request.

## Abbreviations

ADT: Androgen deprivation therapy
AE: Adverse event
CI: Confidence interval
CTCAE: Common terminology criteria for adverse events
DCE: Discrete choice experiment
MFS: Metastasis-free survival
MRS: Marginal rate of substitution
nmCRPC: Non-metastatic castration-resistant prostate cancer
OS: Overall survival
PFS: Progression-free survival
PSA: Prostate-specific antigen
RPL: Random parameter logit
SGARI: Second-generation androgen receptor inhibitor
TPP: Time to pain progression
US: United States
