# Supplementary material for: Physician preferences for non-metastatic castration-resistant prostate cancer treatment
Source: BMC Urol. 2020 Jun 22;20:73. doi: 10.1186/s12894-020-00631-4 (PMC7310549; doi:10.1186/s12894-020-00631-4)
Supplement: Supplementary file 1 — Additional file 1: Appendix Table 1. Labels and Descriptions Used for Efficacy Attributes and Levels [file 12894_2020_631_MOESM1_ESM.docx]

# Appendix Table 1: Labels and Descriptions Used for Efficacy Attributes and Levels

| **Attribute Label** | **Attribute Description** | **Attribute Level Description** | | |
| --- | --- | --- | --- | --- |
| Prolonging life | For this survey, assume that patients with nmCRPC usually live for another 4 years. The medication may help to slow or stop the growth of the prostate cancer such that the patients live a few months longer (for example, 4 years and an additional ___ months). We will be asking you to compare medications which can prolong life such that patients can live for another: | 4 years and an additional 12 months  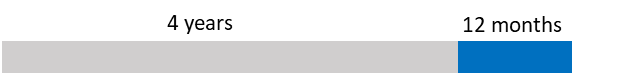 | 4 years and an additional 6 months  **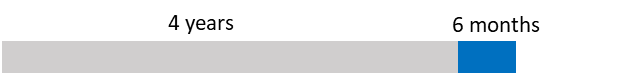** | 4 years and an additional 3 months  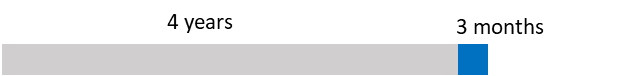 |
| Delay in time until pain progresses (develops or worsens) | For this survey, assume that patients with nmCRPC usually have 3 years until their pain progresses. This means that the patients will develop pain if they are not currently experiencing pain, or they will experience pain that is worse than what they currently have. You think that the progression of pain is clinically meaningful.  The medication can better control the cancer and delay the time until patients’ pain start to worsen by a few more months (for example, 3 years and an additional ___ months). We will be asking you to compare medications that can delay the time until pain progresses by: | 3 years and an additional 12 months  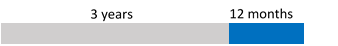 | 3 years and an additional 6 months  **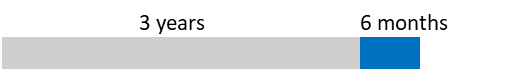** | 3 years and an additional 3 months  **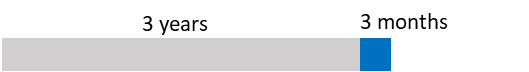** |
