# Supplementary material for: Physician preferences for non-metastatic castration-resistant prostate cancer treatment
Source: BMC Urol. 2020 Jun 22;20:73. doi: 10.1186/s12894-020-00631-4 (PMC7310549; doi:10.1186/s12894-020-00631-4)
Supplement: Supplementary file 2 — Additional file 2: Appendix Table 2. Labels and Descriptions Used for Risk (Adverse Event) Attributes and Levels [file 12894_2020_631_MOESM2_ESM.docx]

**Appendix Table 2: Labels and Descriptions Used for Risk (Adverse Event) Attributes and Levels**

| **Attribute Label** | **Attribute Description** | **Attribute Level Description** | | |
| --- | --- | --- | --- | --- |
|  |  | **None** | **Mild-to-Moderate** | **Severe** |
| Fatigue (lack of energy) | Patients who take the medication may experience fatigue as a side effect. This means that they feel weak and have a lack of energy to carry out their daily activities. The different severity levels of fatigue we will ask you to consider include: | No fatigue | **(may affect daily activities)**: Fatigue can sometimes go away with rest. However, sometimes the fatigue does not go away with rest and may limit patients’ daily activities such as shopping for groceries and doing housework. | **(affects self-care):** Fatigue does not go away with rest and keeps coming back. The lack of energy limits the ability of patients to take care of themselves. For example, they need help getting out of bed or getting dressed. |
| Skin rash | Patients who take the medication may develop a skin rash as a side effect. Symptoms of rash can include itchiness, a burning sensation, feeling of tightness, feeling of tenderness, or blisters. The different severity levels of skin rash we will ask you to consider include: | No skin rash | **(30% of the body or less, may affect daily activities**): Skin rash that is limited to certain areas of the body (30% of the entire body or less). Because of the skin rash, patients may avoid social activities. The skin rash may limit patients’ daily activities such as shopping for groceries and doing housework. | **(more than 30% of the body, require treatment and affects self-care):** Skin rash that is wide-spread and covers a large area of the body (more than 30% of the entire body). The skin rash may be painful and patients may need treatment for the rash. The skin rash can be life-threatening if it becomes infected. The skin rash limits the ability of patients to take care of themselves and they may need someone to take care of them. |
| Cognitive problems | Patients who take the medication may develop cognitive problems as a side effect. These problems can include being confused, having trouble remembering things, concentrating, learning new things, thinking clearly, and making decisions in everyday life. The different severity levels of cognitive problems we will ask you to consider include: | No cognitive problems | **(may affect daily activities)**: Some problems with confusion, remembering things, concentrating, learning new things, thinking clearly, or making decisions from time to time. Patients may face challenges keeping up with their daily activities such as working, managing money, or shopping for groceries. | (**affects self-care):** Serious problems with confusion, remembering things, concentrating, learning new things, thinking clearly, or making decisions. This limits the ability of patients to take care of themselves and they may need someone to help take care of them. |
| **Attribute** | **Attribute Description** | **None** | **5%** | **8%** |
| Risk of a serious fall | Patients who take the medication may have a higher risk of experiencing a serious fall as a side effect. A serious fall results in injuries that require patients to be admitted to a hospital.  The different risks of a serious fall that we will ask you to consider include: | None (0 out of 100 people)  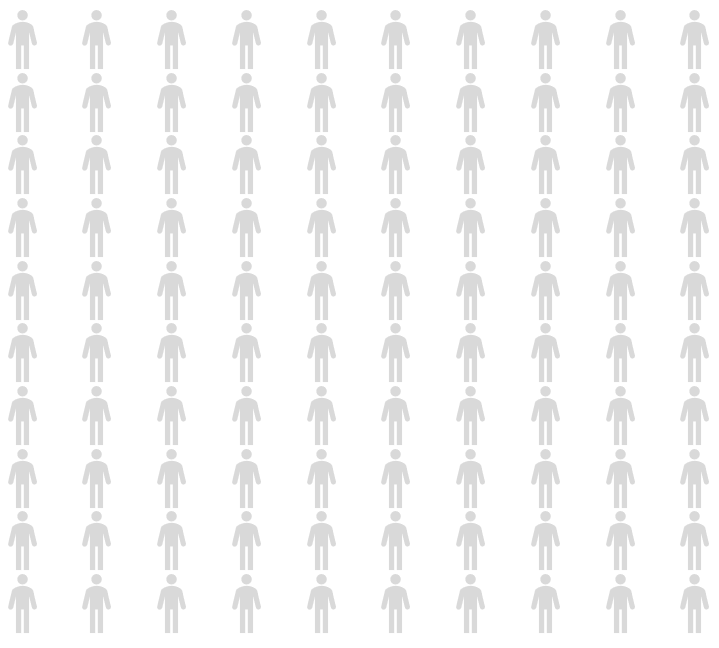 | 5% (5 out of 100 people)  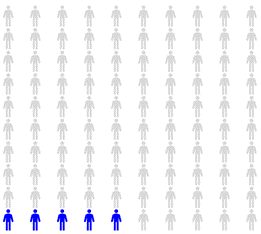 | 8% (8 out of 100 people)  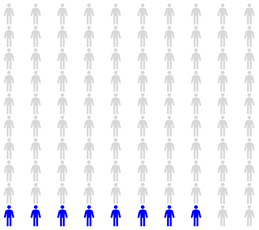 |
| Risk of a serious fracture | Patients who take the medication may have a higher risk of experiencing a serious fracture as a side effect. A serious fracture is disabling (severely limits patients’ movement and/or activities, and which may be permanent). The bone may be dislocated and exposed. Immediate medical care or surgery is needed to fix the serious fracture. This may be life-threatening.  The different risks of a serious fracture that we will ask you to consider include: | None (0 out of 100 people)  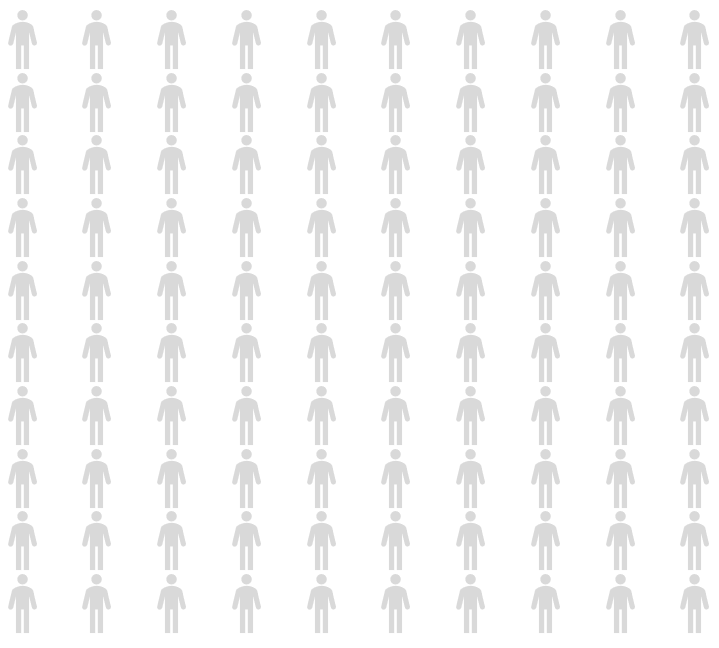 | 5% (5 out of 100 people)  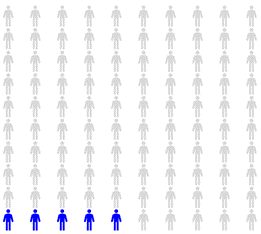 | 8% (8 out of 100 people)  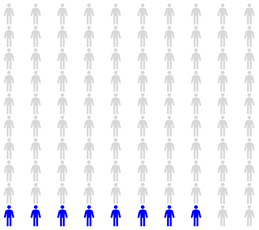 |
