# Supplementary material for: Physician preferences for non-metastatic castration-resistant prostate cancer treatment
Source: BMC Urol. 2020 Jun 22;20:73. doi: 10.1186/s12894-020-00631-4 (PMC7310549; doi:10.1186/s12894-020-00631-4)
Supplement: Supplementary file 3 — Additional file 3. Sample Size Considerations in Discrete Choice Experiments [file 12894_2020_631_MOESM3_ESM.docx]

# Sample size considerations in Discrete Choice Experiments (DCEs)

Sample size calculations in DCE is complicated and depends on the true values of the estimated parameters in the choice model.^^[[1]](#footnote-1)^,^^^[[2]](#footnote-2)^^ Most DCE studies in health care have included sample sizes in the range of 100 to 300.^2,^[[3]](#footnote-3)^^ A common rule of thumb formula used is^^[[4]](#footnote-4)^,^^[[5]](#footnote-5)^:

N>500c/(t x a)

*N = minimum sample size*

*c = largest number of levels for any of the attributes*

*t = number of choice tasks*

*a = number of alternatives*

1. Lancsar E and Louviere J. Conducting discrete choice experiments to inform healthcare decision making: a user's guide. Pharmacoeconomics. 2008;26(8):661-77. [↑](#footnote-ref-1)
2. Bridges JF, Hauber AB, Marshall D, et al. Conjoint analysis applications in health—a checklist: a report of the ISPOR Good Research Practices for Conjoint Analysis Task Force. Value in health. 2011;14(4):403-413. [↑](#footnote-ref-2)
3. Marshall D, Bridges JF, Hauber B, et al. Conjoint Analysis Applications in Health - How are Studies being Designed and Reported?: An Update on Current Practice in the Published Literature between 2005 and 2008. Patient. 2010 Dec 1;3(4):249-56. [↑](#footnote-ref-3)
4. Orme B. Sample Size Issues for Conjoint Analysis. https://www.sawtoothsoftware.com/download/techpap/samplesz.pdf. Accessed July 13, 2018. [↑](#footnote-ref-4)
5. de Bekker-Grob EW, Donkers B, Jonker MF, Stolk EA. Sample size requirements for discrete-choice experiments in healthcare: a practical guide. The Patient-Patient-Centered Outcomes Research. 2015;8(5):373-384. [↑](#footnote-ref-5)
